# Morphology Analysis and Optimization: Crucial Factor Determining the Performance of Perovskite Solar Cells

**DOI:** 10.3390/molecules22040520

**Published:** 2017-03-24

**Authors:** Wenjin Zeng, Xingming Liu, Xiangru Guo, Qiaoli Niu, Jianpeng Yi, Ruidong Xia, Yong Min

**Affiliations:** 1 Key Laboratory for Organic Electronics & Information Displays (KLOEID) & Institute of Advanced Materials (IAM), Jiangsu National Synergistic Innovation Center for Advanced Materials (SICAM), Nanjing University of Posts and Telecommunications, 9 Wenyuan Road, Nanjing 210023, China; iamwjzeng@njupt.edu.cn (W.Z.); 18705196503@163.com (X.L.); gxiangru@gmail.com (X.G.); iamqlniu@njupt.edu.cn (Q.N.); yjpmvp2012@126.com (J.Y.); iamygmin@njupt.edu.cn (Y.M.); 2 The School of Materials and Energy, Guangdong University of Technology, Panyu, Guangzhou 510006, China

**Keywords:** photovoltaic, perovskite, CH_3_NH_3_PbI_3_, morphology, energy alignment, interface

## Abstract

This review presents an overall discussion on the morphology analysis and optimization for perovskite (PVSK) solar cells. Surface morphology and energy alignment have been proven to play a dominant role in determining the device performance. The effect of the key parameters such as solution condition and preparation atmosphere on the crystallization of PVSK, the characterization of surface morphology and interface distribution in the perovskite layer is discussed in detail. Furthermore, the analysis of interface energy level alignment by using X-ray photoelectron spectroscopy and ultraviolet photoelectron spectroscopy is presented to reveals the correlation between morphology and charge generation and collection within the perovskite layer, and its influence on the device performance. The techniques including architecture modification, solvent annealing, etc. were reviewed as an efficient approach to improve the morphology of PVSK. It is expected that further progress will be achieved with more efforts devoted to the insight of the mechanism of surface engineering in the field of PVSK solar cells.

## 1. Introduction

**Figure 1:**
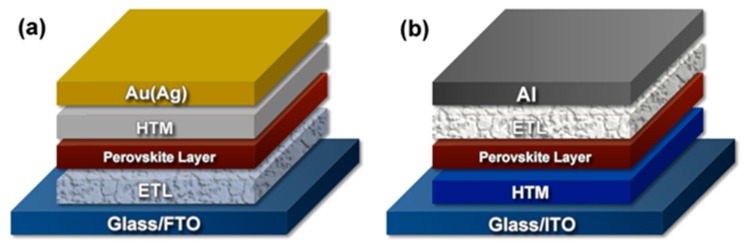
Structure of PVSK solar cells: (**a**) conventional device and (**b**) inverted device.

Perovskite (PVSK) solar cells have attracted increasing research focus recently since they embody promising prospects for massive commercialization. PVSK solar cells possess enormous superior features such as low cost, simplicity of fabrication, high performance, etc. Remarkable progress on PVSK research has been achieved with the power conversion efficiency (PCE) significantly increasing from 3.8% to beyond 22% in the past few years [1,2,3,4,5,6,7,8]. Such progress derives from the synthesis of PVSK materials and charge-transport media, the proposal of new device architecture, as well as the improvement in device fabrication technology and process, etc. In particular, material crystallinity and aggregation distribution, as general reported, play a dominant role in determining the electronic properties in both organic and inorganic electronic materials and the performance of electronic devices [9,10,11,12,13]. As depicted in Figure 1a, the conventional architecture of PVSK solar cells is typically including conducting glass substrate (e.g., the fluorine doped tin oxide (FTO) substrate), electron-transport layer (ETL), perovskite layer, hole-transport medium (HTM), and metal electrode (e.g., Au or Ag). It was generally believed that surface morphology of both PVSK materials and the carrier-transporting materials (including HTM and ETL), has strong dependence on the approaches of the film deposition and the condition of crystalline formation.

In contrast to the conventional device configuration, the inverted cell architecture (Figure 1b), where the perovskite layer is deposited on the hole-transport medium HTM or directly on a contact electrode for holes extraction and then covered by an ETL, has recently attracted attention due to its less pronounced hysteresis in the current–voltage response in corresponding devices. The reported highest PCE is 15% for the inverted devices [14]. The carrier-transport layer of PVSK solar cells can be divided into two kinds, i.e., HTM and ETL, according to its energy level. The most widely-used HTM in PVSK solar cells is 2,2′,7,7′-tetrakis(*N*,*N*-di-*p*-methoxyphenylamine)-9,9′-spirobifluorene (spiro-MeOTAD). However, the high cost and low stability of spiro-MeOTAD limit the commercialization of PVSK solar cells. Therefore, low-cost metal oxide [15,16,17], copper iodide (CuI) [18], and copper thiocyanate (CuSCN) [19,20] were used as HTMs to replace spiro-MeOTAD. Further improvement has been reported on developing a novel and facile method to prepare Cu_2_O and CuO films as HTMs. PVSK solar cells based on such HTM exhibited a high efficiency of 13.35% and improved stability with PCE remaining above 90% of the initial value after stored for 70 days [21]. Recently, polymer-based HTMs have also received increased attention. For example, low-cost poly(3-hexylthiophene) (P3HT) [22], poly(3,4-ethylenedioxythiophene) (PEDOT) and layered V_2_O_5_/PEDOT nanowires were reported to be HTM for PVSK solar cells [23,24,25,26,27,28,29].

The typical ETL materials for PVSK solar cells include TiO_2_ [30], ZnO [31,32], V_2_O_5_ [33], and Al_2_O_3_ [34], etc. With the modification of the nanostructure of such materials, the improvement such as low grain boundaries, effective charge separation and collection ability can be achieved. This has been the research focus for PVSK solar cells. For example, 1-D nanorod [35], 2-D nanosheet [36], and 3-D nano-array [37,38,39,40] of TiO_2_ were investigated as HTM to seek for pathway for enhanced performance. The comprehensive insight of the impact factors of the surface morphology emphasizes that the development of new methods to control and optimize the surface morphology has become a dominant way for the further improvement of the performance and the stability of PVSK solar cells.

Therefore, in-depth study of PVSK solar cell mainly focuses on the characterization of the charge-transport layer and PVSK layer, the alignment of the interface energy level, modification of the surface morphology, and control of the distribution of the materials crystals and aggregates, optimization of the surface properties and so on.

To date, many approaches have been proposed for the modification of surface morphology in PVSK solar cells. This review will focus on the morphology analysis by the common characterization methods of scanning electron microscopy (SEM), optical microscopy, Fourier transform infrared spectroscopy (FTIR), Raman spectroscopy, etc. Based on the characterization of the surface morphology, significant impact factors are figured out to demonstrate the formation process of the charge-transport film and PVSK layer. Furthermore, X-ray photoelectron spectroscopy (XPS) and ultraviolet photoelectron spectroscopy (UPS) of PVSK layers are discussed to explain the correlation between the morphology and interface energy level alignment. Methods for performance improvement of PVSK are also summarized for further promotion towards practical commercialization.

## 2. Surface Morphology and Spectrum Analysis

### 2.1. Fabrication Processions Related Surface Morphology of the Perovskite Layer

The morphology investigation in PVSK solar cells typically includes two topics, i.e., the PVSK materials and carrier-transporting materials (including HTM and ETL).

#### 2.1.1. PVSK Materials

In the past few years, two perovskite materials, i.e., CH_3_NH_3_PbI_3_ (MAPbI_3_) and CH_3_NH_3_PbI_3-x_Cl_x_ (MAPbI_3−x_Cl_x_), have been widely investigated in the solar cell scope. To compare the properties of both materials, much attention has been devoted to their surface chemical composition and, specifically, the surface composition. SEM, including top-view and cross-sectional SEM, is widely used to characterize and analyze the surface morphology of perovskite materials. It can be used to record the grain growth status of the perovskite layer.

**Figure 2:**
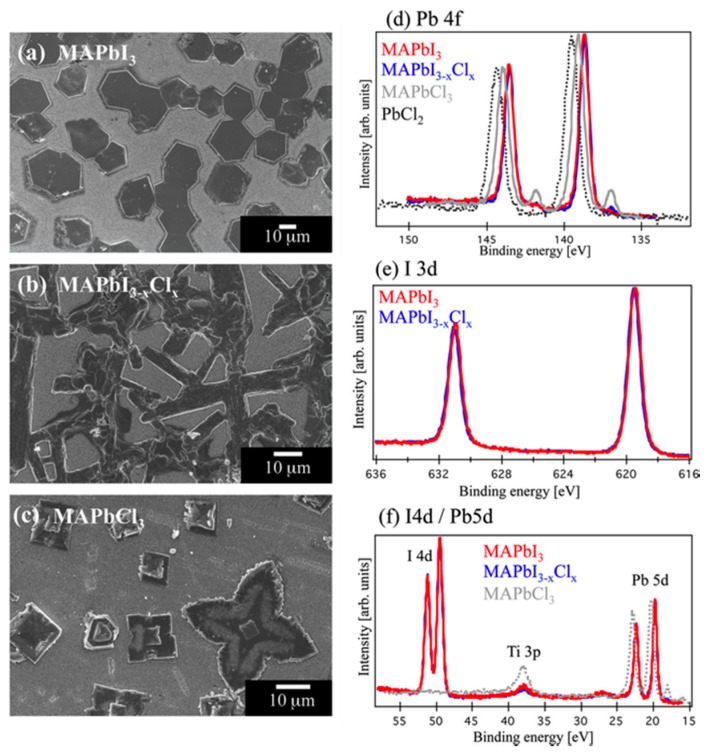
Top-view SEM images of (**a**) the MAPbI_3_ (red frame); (**b**) MAPbI_3−x_Cl_x_ (blue frame); and (**c**) MAPbCl_3_ (gray frame) materials deposited on a TiO_2_/FTO substrate. (Horizontal scale bars = 10 mm.) Also shown are (**d**) Pb 4f spectra; (**e**) I 3d spectra; and (**f**) Pb 5d/I 4d spectra of MAPbI_3_ (red solid line), MAPbI_3−x_Cl_x_ (blue solid line), and MAPbCl_3_ (light gray line) recorded with an excitation energy of 4000 eV [41]. Copyright © 2015, American Chemical Society, Washington, DC, USA.

Figure 2 presents the SEM images of hexagonal MAPbI_3_ samples, a more heterogeneous MAPbI_3−x_Cl_x_, and MAPbCl_3_ along with their corresponding photoelectron spectroscopy [41]. It indicates that chlorine effects the formation of crystalline MAPbI_3−x_Cl_x_ significantly during the film preparation. Detailed examination of annealed MAPbI_3−x_Cl_x_ film reveals that only very low Cl concentration could be incorporated in perovskite MAPbI_3−x_Cl_x_. The difference between using PbCl_2_ and PbI_2_ as the precursor material is significant in terms of crystal morphologies and film coverage, which is related to the growth rate of PVSK crystal and Cl incorporation. When the molar concentration of PbI_2_ (>0.5 M) exceeds that of PbCl_2_, the device parameters (V_oc_, *J*_sc_, FF, and PCE) tend to deteriorate, as tabulated in Table 1 in detail [42]. This rule also applied to other materials systems, such as (FAPbI_3_)_0.85_(MAPbBr_3_)_0.15_, etc. in the recent study [43,44,45,46].

More recently, an in-depth investigation on the crystallization process and mechanism were presented in Ref. [47], with the high PCE above 20% based on FAPbI_3_ as the perovskite layer. It was revealed that the phase of PbI_2_(DMSO)_2_ can be transformed by releasing some DMSO molecules when annealed at a low temperature at 60 °C. The application of TGA testing can clearly determine the content of DMSO in the as-annealed powder. Also the composition of the FAPbI_3_-based layer was accurately determined by XRD analysis. The pseudocubic lattice parameter is determined to be 6.348 Å as for the FAPbI_3_/MAPbBr_3_ film. Therefore, the presented crystallization process, so-called the intramolecular exchange process, can lead to pure FAPbI_3_-based film in high quality, with the preferred orientation along the [111] axis.

#### 2.1.2. Carrier-Transporting Layers

The morphology and crystallinity of the carrier transporting layer are also critical to the performance of PVSK solar cells. It is reported that a systematic investigation of photovoltaic performance and its long term stability of MAPbI_3−x_Cl_x_ based perovskite solar cell have been carried on by varying the morphology and crystallinity of the photoanode material. Three photoanodes were compact TiO_2_ layer employed in planar device, rutile nanorods (NRs), and post-treated NRs (TiCl_4_-NRs) [48]. It was observed that planar device structure was not favorable to the long-term performance of the device. Whereas, long term durable devices can be made by confining the perovskite in the mesoporous films of high crystallinity, for example, in the case of nanorod scaffolds based devices. It has become a common view that TiO_2_ particle size in mesoscopic PVSK solar cells has a substantial impact on the photovoltaic performance of the devices. A study focusing on anatase-TiO_2_ nanoparticles of different particle sizes (5, 30, 60, and 90 nm) was performed and exhibited a trend of changes in photocurrent density and fill factor as well as in the quality of perovskite film [49]. It was observed that the porosities of all the mesoporous films keeps at approximately 30% regardless of the difference in the particles sizes, whereas surface area of the obtained films was decreased with increasing particles sizes. The devices based on the smaller (5 and 30 nm) anatase-TiO_2_ nanoparticles exhibited better fill factor, compared to those of device based on larger (60 and 90 nm) anatase-TiO_2_ nanoparticles. In contrast, larger particles-based devices showed a higher photocurrent values (up to 22 mA/cm^2^). The maximum PCE of 11.1% appeared based on 60-nm nanoparticles [49]. Therefore, many efforts were done to optimize the morphology of the carrier-transporting layer [50,51,52]. For example, unique vertically-aligned TiO_2_ in nanocone-shaped morphology was reported, synthesized via a hydrothermal method. Such morphology enhanced the PCE of PVSK based on MAPbI_3_ by 11% [53].

#### 2.1.3. Film-Deposition Approaches

One of the rapid progresses in the PVSK solar cells is the formation techniques of the perovskite films. Most of the conventional techniques can be divided into two methods, film formation by solution crystallization or vapor crystallization. High efficiency has been achieved by both of the approaches for devices based on some common perovskite materials, such as MAPbI_3_. However, it is interesting to make clear which approach is more desirable for high performance device. This has been elucidated by investigating the influence of crystallization condition on perovskite morphology and its correlated device performances [54].

**Figure 3:**
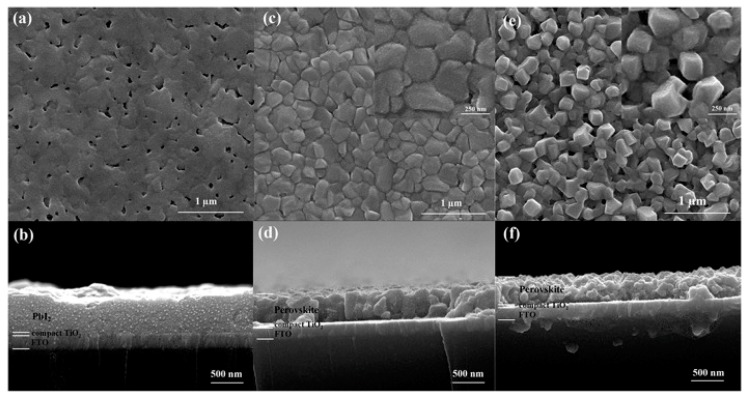
Top-view SEM images of (**a**) PbI_2_; (**c**) vapor-crystallized perovskite; and (**e**) solution-crystallized perovskite; the insets of c and e show magnified images of perovskite crystals. (**b**,**d**,**f**) Corresponding cross-view SEM images of PbI_2_, vapor crystallized perovskite, and solution-crystallized perovskite [54]. Copyright © 2015, American Chemical Society.

Figure 3 shows typical top-view (Figure 3a,c,e) and cross-sectional (Figure 3b,d,f) SEM images of PbI_2_, vapor-crystallized MAPbI_3_ and solution-crystallized MAPbI_3_, respectively. It can be seen that PbI_2_ almost covers the entire area of compact TiO_2_ smoothly with only few small voids, vapor-crystallized MAPbI_3_ perovskite demonstrates smooth surface with compactly aligned perovskite (Figure 3c), while solution-crystallized perovskite consisted of loosely accumulated cubic crystals (Figure 3e) with big voids and pinholes due to the chemical reaction between the CH_3_NH_3_I (MAI) solutions and PbI_2_-coated substrates. The yellow color of PbI_2_ was observed to change into dark brown immediately after dipping MAI solutions. The different preparation techniques of the MAPbI_3_ film could cause the variation in the reaction ratio between PbI_2_ and MAPbI_3_, which lead to different growth mechanism of the perovskite crystals. The vapor-crystallized MAPbI_3_ displayed more vertically oriented crystals than its counterpart solution-crystallized MAPbI_3_. In planar-structured solar cells, electrons must diffuse through the whole perovskite layer. Therefore, vertically oriented crystals are more desirable for electron transportation. Vapor-crystallized devices normally exhibit enhanced absorption, suppressed dark current and lower recombination rate. As a result, much higher PCE of 8.1%, was reported for vapor-crystallized devices with comparison of the efficiency of 5.8% for solution-crystallized devices [54]. Although the PCE results indicated that the vapor-crystallized method was favorable to the solution-crystallized method in terms of obtaining good photovoltaic performance, high device performance could still be attained with optimized solution crystallized technique. A solution route was reported for the deposition of uniform, large-grain, textured, and high-crystallinity planar MAPbI_3_ perovskite films with high-aspect-ratio grain structure [55]. Room-temperature solvent-bathing process was introduced to promote rapid nucleation/crystallization of MAPbI_3_ perovskite from the precursor film to suppress the grain growth. As a result, a maximum PCE of 18.3% for device size of 0.12 cm^2^ has been achieved.

More recently, novel approaches, such as contact-passivation strategy [7], vacuum flash–assisted solution process [56], kinetically controlled gas–solid reaction film fabrication process [57], simplified close space sublimation [58], are reported to further enhance the PVSK’s performance by reducing the pin holes of the perovskite film, overcoming short-circuit current to realize large dimensional devices.

### 2.2. Impact Factors to the Perovskite Morphology

#### 2.2.1. Effect of Humidity

It is a great challenge to control humidity conditions for fabricating high-quality perovskite films due to the sensitivity of the grain size to moisture. It was reported that the influence of ambient humidity on nucleation at spin-coating stage is different from that on crystal growth at annealing stage [15]. Water usually plays a negative role in the device performance, since there is a competition between DMSO and H_2_O molecules via coordination chemistry reactions. When the humidity decreased to 30%, the required amount of DMSO is increased to achieve optimum morphology, since the amount of multiiodide plumbate defect is reduced. However, as the humidity increases to 60%, H_2_O is acting as an additive, therefore a lower amount of DMSO is required to provide large crystalline domains. The presence of hydrates can significantly enhance the conductivity of the perovskite layer by several orders. Hence it can create alternative pathways for the carriers to reach the electrodes. As a result, the selection of the contact materials would be more demanding to avoid shunting. Therefore, the mixture ratio of H_2_O/DMSO should be more carefully to reduce the chemical defects and achieve high device performance when the fabrication process is performed under ambient atmosphere [59].

**Figure 4:**
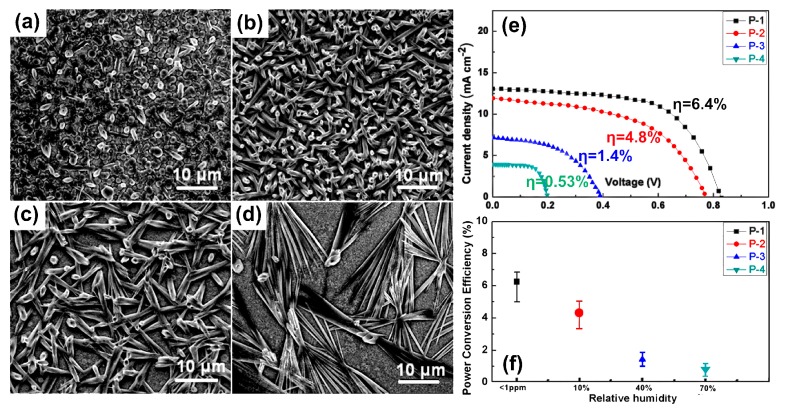
SEM images of perovskite MAPbI_3_ films deposited on mp-TiO_2_ layer under different ambient humidities, i.e., (**a**) <1 ppm (P-1); (**b**) 10% (P-2); (**c**) 40% (P-3) and (**d**) 70% (P-4); (**e**) J–V curves of the perovskite solar cells prepared under different ambient humidities of MAPbI_3_; (**f**) Dependence of the PCEs on RH. Each data point represents the mean from a set of 10 devices [60]. Copyright © 2015, American Chemical Society.

Figure 4 shows the morphology of MAPbI_3_ based perovskite films deposited on mesoporous TiO_2_ layer is significantly influenced by the ambient humidity. As the relative humidity (RH) less than 1 ppm, the coverage of MAPbI_3_ film is high and the shape of the crystallites is much planar (Figure 4a). As the RH is increased to ~10%, the MAPbI_3_ film appears different with the surface coverage of MAPbI_3_ film to the substrate decreases although the micro-particles still in connection with each other (Figure 4b). The film formed under RH ≈ 40% is similar to that formed under RH ≈ 10% in appearance, but the spaces between the perovskite crystallites become larger (Figure 4c), resulting in further reduction of the coverage of perovskite layer. As RH increased to 70%, the perovskite crystallites adopted a larger individual size for islands (Figure 4d). These island-shape crystallites have large sizes of dozens of micrometers and larger spacing in between, resulting in the largest uncovered mesoporous TiO_2_ surface among these four cases. It’s concluded that high RH always induced large crystal islands of perovskites and the relatively large spacing in between, leading to low surface coverage. Similar studies on coverage of the perovskite layer also reported that the high coverage of perovskite layer under low RH resulted in lower light transmittance, therefore, higher PCE of perovskite cells due to better light absorption [60,61,62,63].

Due to the hygroscopic nature of the methylammonium component, humidity of the environment during the fabrication process plays an important role on the formation of methylammonium lead halide perovskite films, which impact the performance of device in both negative and positive ways. The films formed in higher humidity atmospheres demonstrated lower coverage of the perovskite materials. However, such morphology was reported to be advantageous for its application in device, i.e., resulting in significant improvement in open-circuit voltages and the overall performance of device [64].

It has been shown that perovskite exposure to H_2_O does not simply cause the reversion from MAPbI_3_ to PbI_2_. Instead, H_2_O reacts with the perovskite to form a hydrate product similar to (CH_3_NH_3_)_4_PbI_6_·2H_2_O in the dark. This causes a distinct change in the crystal structure of the material. As a result, light absorption spectrum demonstrated a clear decrease in the visible region. The deleterious effects of humidity on completed solar cells, specifically on photovoltaic efficiency and stability, are observed [65]. Before humidity exposure, all of the perovskite films have a rough surface. However, after being in 90% RH for 14 days, the perovskite undergoes a recrystallization process, becoming smooth and highly ordered. Films stored under 0% and 50% RH show similar, but less long-range ordered, structural changes over this time period, exhibiting a coarsening of the perovskite, similar to the report for MAPbI_3−x_Cl_x_ films stored under Ar. This study suggests a promising approach to improve moisture resistance by strengthening the hydrogen-bonding interaction between the organic cation and metal halide octahedra and/or weakening the hydrogen interaction between the organic cation and H_2_O. The excellent stability of device is highly demanded for PVSK solar cells eventually toward commercialization since the commercial devices have to be operated at the ambient atmosphere after normal encapsulation.

#### 2.2.2. Effect of Solutions

Solution deposition of planar films of the pure hybrid perovskite materials, generally results in small grain size high density of defects and low surface coverage. Such poor morphology will cause low power conversion efficiency. Among various fabrication processions, the equilibrium post-deposition treatment, vapor-equilibrated re-growth (VERG), was reported feasible to control the micron size of MAPbI_3_ grains [66]. This approach succeeded to attain pure and homogeneous iodide MAPbI_3_ with large grain sizes and the lifetime of minority carriers longer than 200 ns in the films.

Although PVSK solar cells with the highest PCE beyond 22% have been reported based on MAPbI_3_ [67], it’s still a challenge to form a homogeneous pinhole-free perovskite film, which is crucial to realize reproducible high-efficient PVSK solar cells [68]. The solvent for the MAI precursor and the lead halide perovskite play a dominant role in the formation of the homogenous perovskite layer. It was reported that polar solvent, such as ethanol, is superior to low-polar ones, such as isopropanol. PVSK solar cells processed from ethanol solution demonstrated an enhanced short-circuit current density of 17.31 mA/cm^2^ and a higher fill factor of 77.2%, with a corresponding PCE of 11.45%. In contrast, the devices processed from isopropanol solution exhibits a lower *J_SC_* of 14.38 mA/cm^2^, a FF of 64.8%, and a PCE of 8.21% [69]. Therefore, it is clear that the morphology of PVSK crystalline can be artificially controlled by solution methods.

Low dimensional MAPbI_3_, such as one-dimensional (1D) nanostructure is more desirable than 3D structure since it improves the holes migration from perovskite to HTL and allows more efficient charge separation at HTL/perovskite interface. A two-step spin-coating procedure was reported to prepare organolead iodide perovskite MAPbI_3_ in the form of nanowire by means of a small quantity of aprotic solvent [70]. Nanowire CH_3_NH_3_PbI_3_ with an average diameter of 100 nm was successfully grown at the aid of aprotic solvent DMF in two-step spin coating procedure. At low concentration of 0.019 M MAI solution, less dense needlelike nanowires film formed, while dense netlike nanowires formed at the solution with MAI concentration ranging between 0.038 M and 0.057 M. The spaces among the nanowires started to be filled as the concentration of MAI higher than 0.076 M. The holes injection from perovskite to spiro-MeOTAD has been greatly improved in nanowire structure compared with in the bulk MAPbI_3_. Therefore, a PCE of 14.71% was achieved for the nanowire perovskite solar cell device. It was also reported that the nanocrystals size and photoluminescence peak of the perovskite film could be tuned by varying the concentration of perovskite in the matrix material via a simple and low-temperature route [71].

The concentration of the precursor solution also affects the morphology of the perovskite films. Mechanistic insights on the crystal growth of lead halide perovskite reveals that MAPbI_3_ conversion growth fellows one of the two possible pathways, an interfacial reaction process or a dissolution-recrystallization process, depending on the MAI precursor concentration. Single crystals, such as nanowires, nanorods, and nanoplates, of methylammonium lead halide perovskites (MAPbI_3_ and MAPbBr_3_) can be successfully grown by elaborately monitoring the MAI concentration and the interface reaction time [72].

**Figure 5:**
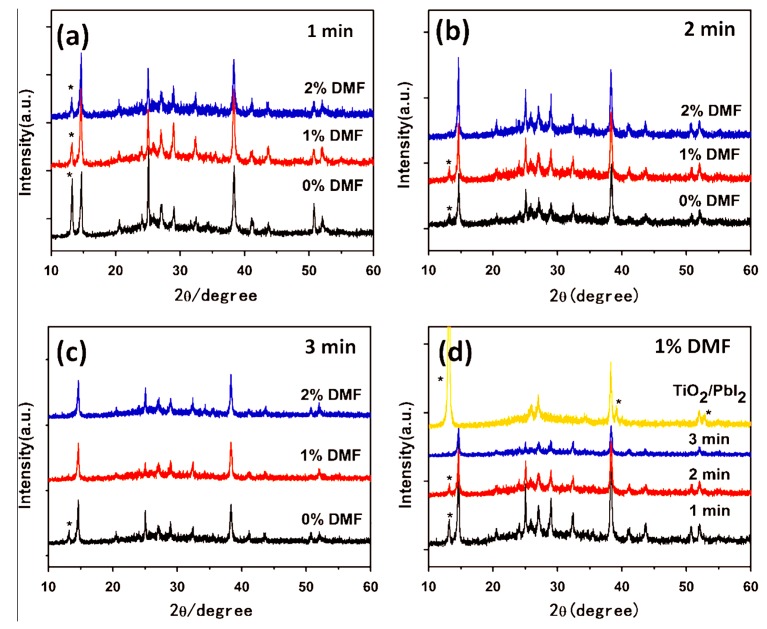
Effect of the ratio of DMF and dipping time on XRD patterns (**a**) 1 min; (**b**) 2 min; (**c**) 3 min; and (**d**) different dipping time with 1% DMF [76]. Copyright © 2015, Elsevier.

The formation of high quality perovskite needs a compact PbI_2_ precursor film. However MAI is difficult to inject into a compact PbI_2_ film, rendering the unreacted and remnant PbI_2_ on the final perovskite film [73,74,75,76]. To facilitate a rapid conversion of PbI_2_ to MAPbI_3_, an efficient method is introduced with the solvent of the dimethylformamide (DMF) to assist the growth of the perovskite films, resulting in the improvement of the photovoltaic performance of PVSK solar cells. The effects of DMF ratio in MAI mixed solution and soaking time on the optical/structural properties of perovskite films have been investigated. Figure 5 indicates the X-ray diffraction (XRD) spectra of MAPbI_3_ prepared from different ratio of DMF and dipping time, which indicates the tetragonal MAPbI_3_ perovskite structure formed and crystal size increase gradually. It has been found that the incorporation of DMF in MAI solution activates and roughens the compact surface of PbI_2_ films due to the good solubility of PbI_2_ in DMF, which leads to a high conversion rate of PbI_2_ to MAPbI_3_, as well as the increases of the crystal size of the perovskite. The interface between perovskite and HTM can be optimized by decreasing the amount of the grain boundaries. This could result in the improvement of the device performance by increasing photocurrent generation [76]. Further experiment also reveals that species with iodides ions in a low number can be formed with the application of the strongly coordinating solvent. All the plumbate ions may act as structural defects, which can influence the electronic properties of the perovskite photovoltaic films [77]. Also the aging time can affect the crystalinity of the PVSK thin film. Once the solution is aged for more than 24 h, the performance of PVSK devices can be significantly improved, due to the better crystalinity and the formation of grain-like feature of the perovskite layer [78].

#### 2.2.3. Effect of Pressure

The crystal structure of the perovskite materials varies with the crystal growth condition during the film formation, while the crystalline and grain size could significantly influence the device efficiency [79,80]. Hydrostatic pressure is an efficient technique for novel functional material design and fundamental research to investigate the pressure effect on the perovskite materials. The phase stability of the organolead bromide perovskite, MAPbBr_3_, has been investigated under hydrostatic pressure up to 34 GPa at room temperature. It was reported that two phase transformations happened below 2 GPa (from *Pm*$\bar{3}$*m* to *Im*$\bar{3}$, then to *Pnma*) and a reversible amorphization started from about 2 GPa, which could be attributed to the tilting of PbBr_6_ octahedra and destroying of long-range ordering of MA cations, respectively.

**Figure 6:**
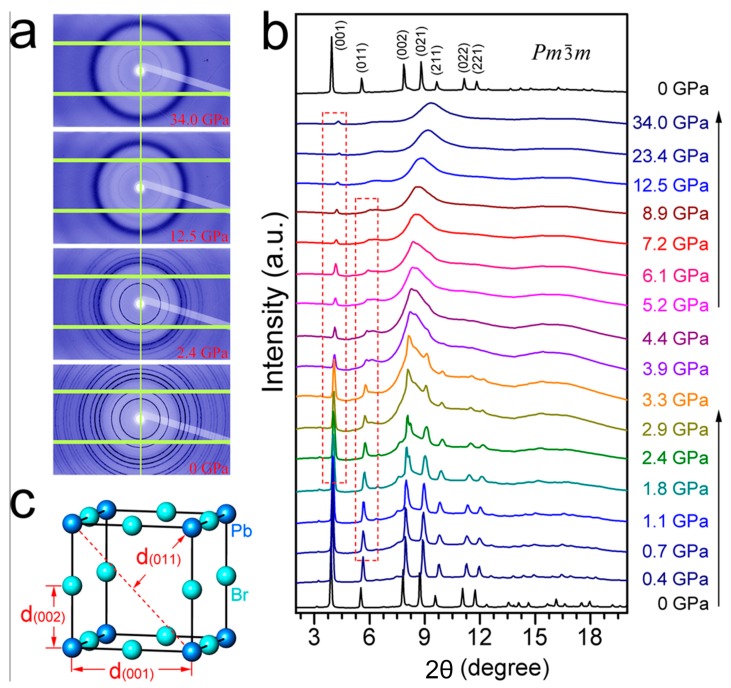
Synchrotron XRD patterns of MAPbBr_3_ obtained during compression up to 34.0 GPa and decompression: (**a**) The raw 2D XRD images and (**b**) integrated 1D XRD profiles. The XRD pattern after decompression can be indexed with the same crystal structure (space group *Pm*$\bar{3}$*m*) from the pristine materials; (**c**) Illustration of the representative interplanar distances in MAPbBr_3_ lattice (Only Pb and Br atoms are drawn for clarity) [81]. Copyright © 2015, American Chemical Society.

Figure 6 indicated the synchrotron XRD patterns of MAPbBr_3_ obtained during compression up to 34.0 GPa and decompression. It was also reported that its maximum electrical resistance reached five orders of magnitude higher than the starting value [81]. The results prove that hydrostatic pressure can greatly affect the crystal structure of organolead halides, which directly cause the change of the photovoltaic related properties, such as a significant enhancement of electrical resistance.

### 2.3. Spectra Analysis of the Perovskite Layer

#### 2.3.1. Fourier Transform Infrared Spectroscopy

Besides scanning electron microscopy investigation of the morphology, spectra analysis techniques are also widely applied in the research of PVSK solar cells. Fourier Transform infrared spectroscopy (FTIR) is one of the spectral analysis techniques used to characterize the structure of compound by analyzing the types of functional groups and the chemical environment of the samples. It is an efficient method to analyze the growth process of the perovskite layer or the carrier transport layers. For examples, one of the reports was published on the application of FTIR to unambiguously detect the modifications of the spiro-MeOTAD and the MAPbI_3_ layers during the drying processes in different gas environments [82]. The FTIR spectra of the powder extracted from the oxidized Spiro-MeOTAD samples on glass substrates indicates that the intensities of peaks at 1136 cm^−1^ were strongly quenched after samples stored to dry air for 12 h, with the powder was stored under different atmospheres in dark for 12 h before the spectra taken. The quenching of these vibrational modes is speculated to be the removal of Li^+^ ion from Li-TFSI molecule, which is operated by the oxygen radical anion and to the rearrangement of spiro-MeOTAD and TFSI molecules [83]. It suggests that dry air is the most promising storage ambient during the drying process of spiro-MeOTAD, compared with the atmosphere of vacuum and dry nitrogen. Further absorption measures of the perovskite layer indicates that the enhancement of the PCE of the devices arise from the improved carrier collection at the interface between perovskite/hole-transport material, instead of the optical properties.

#### 2.3.2. Raman Spectroscopy

Raman spectroscopy is sensitive to the structural changes in the molecular framework. Micro-Raman spectroscopy is able to provide laterally resolved microstructural information for a broad range of materials. Raman spectra have also been reported to analyze the change in the local structure of I, Br, and Cl in the perovskite layers [84], as well as to examine the dependence of photovoltaic properties of MAPbI_3_ PVSK solar cells on structural and physicochemical properties of *p*TiO_2_ [85]. Raman spectra of organic—inorganic halide perovskite thin films, including MAPbI_3_, MAPbBr_3_ and MAPbI_3−x_Br_x_, were presented and compared respectively [86].

**Figure 7:**
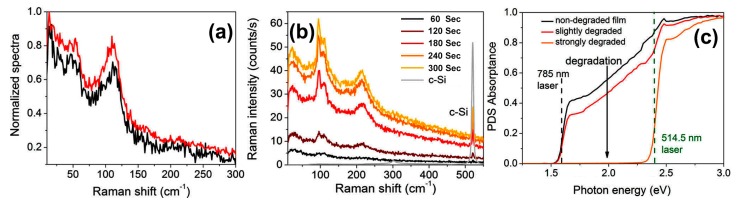
(**a**) Normalized Raman spectra measured at two different positions on a pristine MAPbI_3_ layer using a very low excitation laser power of 10 μW at 514.5 nm; (**b**) Five Raman spectra measured consecutively without any additional delay time at the same position of a MAPbI_3_ layer at the excitation wavelength of 514.5 nm in ambient conditions; (**c**) PDS absorption spectra of MAPbI_3_ layers at various degradation states. The green and black lines indicate the excitation laser wavelengths used for these experiments (514.5 and 785 nm, respectively) [86]. Copyright © 2015, American Chemical Society.

Figure 7a shows the Raman spectra taken at two different positions on a pristine MAPbI_3_ layer at a very low excitation laser power (10 μW at 514.5 nm). Figure 7b depicts the Raman spectra evolution induced by repeated measurement of the same MAPbI_3_ layer deposited on a c-Si wafer with the acquisition time of 60 s for each spectrum. The Raman spectra reveal two distinct bands at 52 and 110 cm^−1^ become stronger with samples degraded. The spectra unambiguously reveal that the final degradation products contain pure PbI_2_, degraded from MAPbI_3_. Figure 7c is the absorption spectra by the photo-thermal deflection spectroscopy (PDS) of MAPbI_3_ layers at various degradation states, which shows the absorption of MAPbI_3_ layers significantly reduced at wavelengths longer than 500 nm (photon energy below 2.4 eV) as the layers degraded [86].

As mentioned above, characterization and analysis of the surface morphology have been proved very important in obtaining high performance of the perovskite solar cell. It is equally important to achieve the match of interface energy between each layer in the devices. One of the aims of surface modification is to realize interface energy level alignment, which will directly determine the generation and collection of the photo-induced charges.

## 3. Interface Energy Analysis and Alignment

### 3.1. Characterization by X-ray Photoelectron Spectroscopy

**Figure 8:**
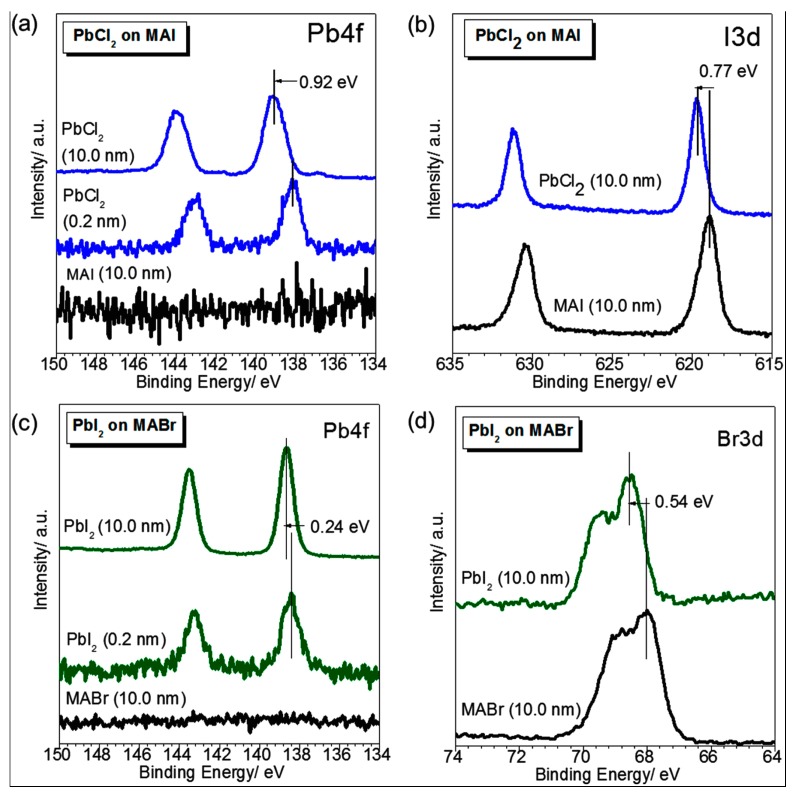
High-resolution XPS core level spectra of (**a**) Pb 4f and (**b**) I 3d when different thicknesses of PbCl_2_ are deposited on 10.0 nm of MAI. XPS core level spectra of (**c**) Pb 4f and (**d**) Br 3d when different thicknesses of PbI_2_ are deposited on 10.0 nm of MABr [88]. Copyright © 2015, American Chemical Society.

Characterization of X-ray Photoelectron Spectroscopy (XPS) provides sufficient evidences for the light absorption, exciton dissociation, and photocharge generation of the perovskites, which are closely related to the strong ionic charge transfer interactions between Pb^2+^ and X^a−^ ions in the perovskite lattices. Figure 8 provides the high-resolution XPS core level spectra near the MAI /PbCl_2_ interface (Figure 8a,b) and MABr /PbI_2_ (Figure 8c,d) when different thicknesses of PbCl_2_ (or PbI_2_) are deposited on top of 10.0 nm of MAI. (or MABr). These XPS results suggest that the MAA^+^ ion is less active in the reaction between MAX^a^ and ${\mathrm{PbX}}_{2}^{b}$, which corroborates with the conclusion that the energy levels of the MAA^+^ cation are located far away from the valence band maximum (VBM, 5 eV below) and conduction band minimum (CBM, 2.5 eV above) of the perovskite film [87,88,89,90].

In some cases, the devices are designed with phenyl-C_61_-butyric acid methyl ester (PCBM)/ZnO as double ETL. The role of the additional ZnO ETL can be studied by XPS and secondary ions mass spectroscopy. It was found that the ZnO layer plays a dual role in the architecture, improving the energy level alignment at the cathode and blocking the reactions between the Al electrode and the perovskite components in air [91].

### 3.2. Characterization by Ultraviolet Photoelectron Spectroscopy

**Figure 9:**
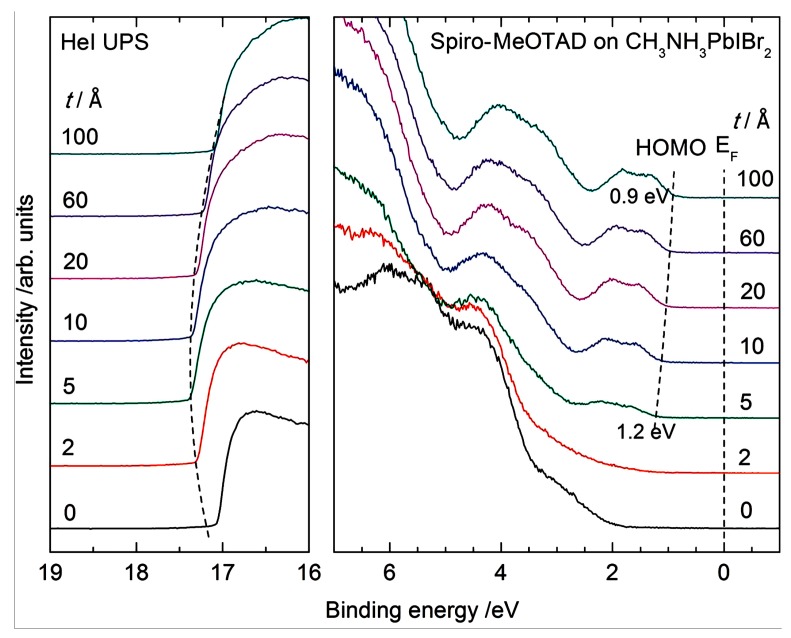
Thickness (*t*) dependence of UPS spectra of spiro-MeOTAD on MAPbIBr_2_. The left panel displays the SECO region and the right panel displays the HOMO region, respectively [94]. Copyright © 2015 WILEY-VCH Verlag GmbH & Co. KGaA, Weinheim, Germany.

UPS is a method to determine the relevant information about the molecular energy level by measuring the energy distribution of the photoelectron excited by the ultraviolet light. It is found that spiro-MeOTAD deposition on perovskite substrates leads to the vacuum level (VL) shift toward higher binding energy at the initial deposition stage, while the progressive VL shift back to lower binding energy with increasing spiro-MeOTAD layer thickness. UPS is the most suitable method to characterize the energy shift. Figure 9 indicates the thickness dependences of UPS spectra of spiro-MeOTAD on MAPbIBr_2_. The way of the VL shift is in accordance with the research outcome that the change of molecular orientation from the initial monolayers to multilayers may induce the VL shift [92,93,94]. It is concluded that the energy level alignment between the perovskite and the HTM can facilitate efficient hole extraction with minimized recombination loss and high electron blocking capability. Therefore HTMs such as spiro-MeOTAD can form a staggered gap heterojunction. In contrary, the HTMs with deep-lying HOMO levels lead to an electron–hole recombination region, which function as a hole blocking layer [94]. Furthermore, XPS was also reported to analyze the chemical and electronic properties of MAPbI_3−x_Cl_x_ perovskite films either spin-coated on mesoporous alumina or evaporated on Si substrates. The study reveals that the elimination of chloride lead to residual methylamine molecules (CH_3_NH_2_) trapped within the perovskite crystal lattice. Such molecules play a role in determining the complex defect mechanisms on the electronic behavior of MAPbI_3−x_Cl_x_ perovskites [95].

As for the inverted device architecture, Ultraviolet spectroscopy and inverse photoemission spectroscopy (UPS and IPES) have also been employed to study the quantitative bulk and interface energetics of the functional materials. The motivation was to improve device characteristics through judicious choosing the transport layer materials in inverted PVSK solar cells [95].

**Figure 10:**
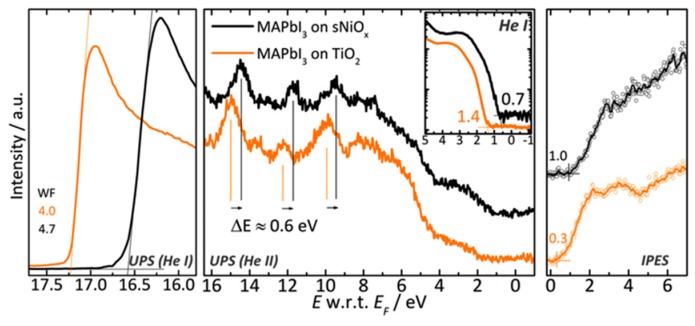
UPS and IPES spectra of MAPbI_3_ on sNiO_x_ (black curve) and MAPbI_3_ on TiO_2_ (orange curve). The left panel shows the secondary electron cutoff for work function determination. The middle panel shows the He II valence band spectra. The VBM measured with He I is shown in inset. The right panel shows the IPES spectra for the determination of the CBM [95]. Copyright © 2015 WILEY-VCH Verlag GmbH & Co. KGaA, Weinheim, Germany.

Figure 10 shows the UPS and IPES spectra of MAPbI_3_ on sNiO_x_ and on TiO_2_, respectively. The onset of the C_60_ HOMO level is steady at 1.3–1.4 eV below *E*_F_ for every layer thickness. On the other side of the bandgap, the onset of the LUMO level measured via IPES is at 0.9 eV above *E*_F_. These combined measurements yield an edge-to-edge HOMO–LUMO gap of 2.2–2.3 eV. Such results indicates that the perovskite layer itself becomes slightly p-doped when deposited on top of the NiO_x_ film, as opposed to the common n-type doping characteristic on top of TiO_2_, hence a unique composition of free carriers and gap states established in MAPbI_3_.

## 4. Approach for Morphology Control and Optimization

### 4.1. Effect of Additives

One of the key points to control the morphology of the perovskite layer is to understand and control the phases and the overall impurity formation in mixed halide perovskite systems. These are crucial for growing high quality crystals and creating reproducible solar cell devices. Solid iodine was applied as a precursor additive to prepare purified organometallic perovskite crystals. The iodine proves to push the chemical reaction toward pure iodine phase rather than the kinetically favored chlorine phase. As a result, the PCE of PVSK solar cells were improved with the average efficiency from 9.83% to 15.58% [96].

**Figure 11:**
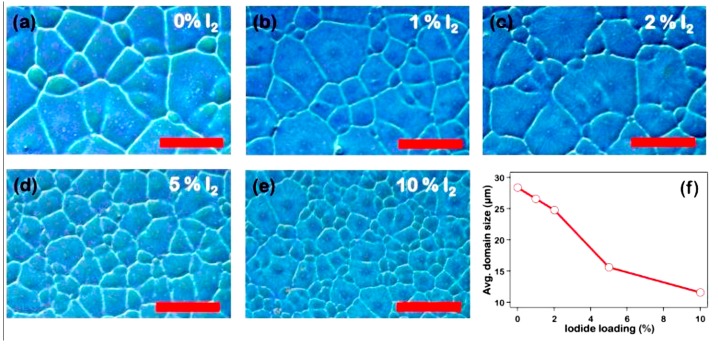
Optical micrographs of hybrid perovskite film (scale bar: 50 μm) from precursor with (**a**) 0%; (**b**) 1%; (**c**) 2%; (**d**) 5%; and (**e**) 10% iodine solution (40 mM). (**f**) Average domain size as a function of iodide loading during fabrication. The average domain size decreases as the iodide loading increases [96]. Copyright © 2015, American Chemical Society.

Figure 11 indicates the optical micrographs of hybrid perovskite film from precursor with the iodine solution at various concentrations. It clearly illustrates the effect of iodide concentration on the film morphology. By adding a small amount (up to 2 vol %, Figure 11b,c) of I_2_-DMF solution, the grain sizes of the perovskite film vary from 56 to 7 μm. With up to 5–10 vol % I_2_ in the solution (Figure 11d,e), the crystal grains appears greater in quantity and smaller in size as compared with that of the crystals from the solution without I_2_. This result suggests that the conversion of MAPbCl_3_ to MAPbI_3_ leads to smaller grain size.

Other additives, such as poly(ethylene glycol) [97], *N*,*N*-dimethyl sulfoxide (DMSO) [98,99], phosphonium halides [100] and low-volatility NH_4_Cl [101], have also been reported to improve the device performance by retarding the growth and aggregation of perovskite crystals, improving the charge mobility, and assisting the crystallization of the perovskite materials, respectively.

### 4.2. Solvent Treatment

**Figure 12:**
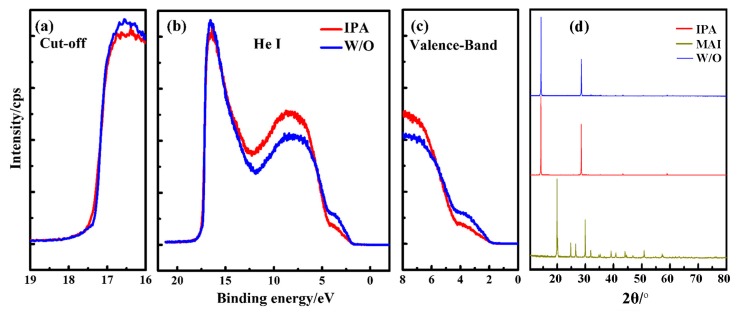
Ultraviolet photoelectron spectroscopy (UPS) of perovskite film with (red line) and without (blue line) IPA treatment on ITO glass substrates, i.e., (**a**) The secondary electron cut-off; (**b**) the full UPS spectrum using He I radiation; (**c**) the valence-band region; and (**d**) X-ray diffraction of perovskite film with and without IPA treatment on ITO glass substrates [113]. Copyright © 2015, Elsevier.

Simple treatments of solvent washing or solvent annealing have been proved efficient to smooth the rough surface of perovskite films [102,103,104,105,106]. The polarity of the solvent has been reported to plays an important role in the effect of the solvent on surface modification. As for non-polar solvent, detailed investigation was performed by comparing the device performance with and without an in-situ treatment of toluene washing to reveal the correlation between the surface morphologies and the crystallizations of perovskite films [107]. The experiment results suggest that in-situ toluene treatment balance the competition between nucleation and crystal growth during the formation of perovskite film on a PEDOT:PSS surface, which leads to a dense perovskite absorber and efficient exciton dissociation at the interface between the perovskite and the PEDOT:PSS [108]. As for polar solvent, ethanolamine [107], chloroform [109], 1,8-diiodooctane (DIO) [110], chlorobenzene [111], different thiols [112], and isopropanol (IPA) [113] were also reported on the surface modification of the perovskite layers. Figure 12 shows the UPS and XRD spectra of perovskite films with and without IPA treatment to study the surface electronic energy levels. The X-ray diffraction study demonstrated that many small peaks observed for the film without treatment disappeared after surface modification treatment in IPA. The appearances of strong peaks at 2θ = 14.2°, 28.4°, 43.3° and 58.9°, corresponding to the (110), (220), (310) and (440) planes, indicates the formation of the tetragonal perovskite structure. This study exhibits eloquent evidence for the conclusion that IPA removes the residues of MAI in MAPbI_3__−__x_Cl_x_ and provides the perovskite films with modified interfacial energy, leading to better electrical conductivity [113].

Post-annealing of solvents is another efficient solvent treatment to induce reassembly of the perovskite crystals for better organization of the film aggregation [114]. Different solvent vapors were observed to have a various but strong effect on the crystal growth of the perovskite layer. The detailed photovoltaic parameters are summarized in Table 2 for the devices post-annealed under different atmospheric conditions. It can be seen that among the listed solvents, PVSK solar cells based on the DMSO-treated films demonstrate an average high PCE over 12% with negligible photocurrent hysteresis [115].

Among the four solvents, i.e., H_2_O, γ-butyrolactone (GBL), dimethylformamide (DMF) and dimethyl sulfoxide (DMSO), DMSO has the lowest volatility due to its low vapor pressure, with the vapor pressure of 17.5, 1.5, 2.7 and 0.42 mmHg at room temperature (20 °C) for H_2_O, GBL, DMF and DMSO, respectively. Therefore the atmosphere of DMSO vapor can be maintained for a longer time, leading to remarkable film growth and crystalline formation. As a result, the DMSO-treated PVSK exhibits the highest device performance [115].

### 4.3. Thermal Annealing

Thermal annealing is another broadly-applied technique to increase the crystallinity of perovskite films. The reported methods of thermal annealing include pre-annealing [116], post-annealing [117], low-pressure vapor annealing [118], multi-step annealing [119], multiple annealing [120,121], and so on.

**Figure 13:**
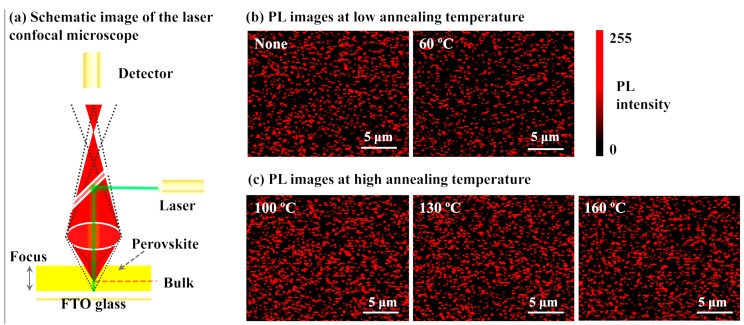
(**a**) Schematic image of the laser confocal microscopy and (**b**,**c**) the measured spatial PL images from the perovskite films annealed at low and high temperatures, respectively [117]. Copyright © 2015, American Chemical Society.

Figure 13 indicates the schematic image of the laser confocal microscopy and the measured spatial photoluminescence (PL) images from the perovskite films annealed at low and high temperatures, respectively. The number of the emission sites increase obviously with the increase of the annealing temperature, which suggests that lower density of non-radiative trap states exist in perovskite films annealed at high temperature than that at low temperature. The variation of the PL intensity for the perovskite films with different annealing temperature could be derived from the modification of the radiative trap states. The reduction of radiative and non-radiative trap states in perovskite film annealed at high temperature is speculated to be associated with less unintentionally doped carriers in the corresponding film [117].

### 4.4. Other Process Optimization

**Figure 14:**
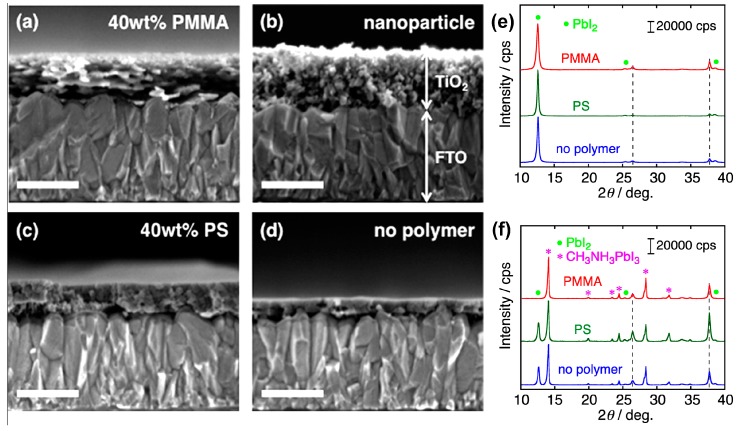
Cross-sectional SEM images of (**a**) TiO_2_ (40 wt %-PMMA); (**b**) TiO_2_ (nanoparticle); (**c**) TiO_2_ (40 wt %-PS); and (**d**) TiO_2_ (without polymer) on FTO/cTiO_2_ substrates. Scale bars are 200 nm, and Wide-angle XRD patterns of (**e**) PbI_2_ and (**f**) perovskite MAPbI_3_ films deposited on FTO/cTiO_2_/TiO_2_ (40 wt %-PMMA) (red line), FTO/cTiO_2_/TiO_2_ (40 wt %-PS) (green line), and FTO/cTiO_2_/TiO_2_ (no-polymers) (blue line). Symbols of green solid circle (•) and pink star (*) represent the XRD diffraction peak position of PbI_2_ and CH_3_NH_3_PbI_3_ [128]. Copyright © 2015, American Chemical Society.

In addition to the mainstream methods of solvent annealing and thermal annealing, other methods in process optimization are also reported for the improvement of PVSK solar cells, such as the application of nonstoichiometric MAPbI_3_ precursor [57], the electrodeposited PbO for the direct conversion to MAPbI_3_ [122], the electrodeposited ultrathin TiO_2_ as the blocking layers [123], layer-by-layer growth of continuous perovskite thin films using an airbrush pen [124], the nanostructured electrode of p-Type NiO formed by a pulsed laser deposition [32], the technique of atomic layer deposition for the perovskite layer [125], the UV-curing-assisted formation of p-type organic electrode interlayer to resist the solvation of the polar precursor solution in fabricating MAPbI_3_ [126], vacuum-vapor assisted solution processing in ambient atmosphere [127], and so on. A novel approach especially mentioned here is utilizing a general polymer, poly(methyl methacrylate) (PMMA), as a unique templating agent for forming crack-free mesoporous TiO_2_ films by a sol-gel method. Figure 14 demonstrates the cross-sectional SEM images of TiO_2_, as well as the wide-angle XRD patterns of PbI_2_ and MAPbI_3_ with and without PMMA as the templating agent on FTO/cTiO_2_ substrates. It can be observed that the simple addition of the general polymer for the sol-gel procedure was able to create the mesoscopic structure for TiO_2_ [128]. Moreover, the crystalline phase of PbI_2_ and MAPbI_3_ on the substrate of FTO/cTiO_2_ proved to be an anatase morphology as for TiO_2_ phase according to the comparison of the wide-angle X-ray diffraction (XRD) pattern. The high polarity of PMMA improved the miscibility with titanium reagents, inducing the meso-sized phase separation. The prompt occurrence of depolymerization in the PMMA domains may also contribute to the nondestructive formation of the mesopores [129]. Therefore, PVSK solar cells prepared by such method exhibiting a PCE_max_ beyond 14%, which is about three times higher than that using a TiO_2_ layer prepared by the same sol-gel method without the polymer addition.

## 5. Summary and Outlook

In summary, the review is to look through some recent investigation on the relationship between the surface morphology and the performance of PVSK solar cells. It focuses on the characterization and the analysis from some commonly used experimental technology, such as SEM, optical microscopy, FTIR, Raman spectroscopy and so on. Key impact factors, e.g., humidity, solution recipe, and pressure of fabrication atmosphere, were realized to play an important role on the formation process of perovskite crystals. Based on the surface morphology analysis, the key issues of interface energy level alignment were discussed by the methods of XPS and UPS. It was pointed out that desirable perovskite film morphology can be attained by architecture optimization and fabrication technique improvement including the blending of additive, solvent annealing, thermal annealing, etc. Based on the rapid progress of PVSK solar cells research and the current high PCE of ca. 20%, it is reasonable to speculate further enhancement of the efficiency and the improvement of the stability with development of the surface engineering. This will lead to the prospect of the massive commercialization of the perovskite solar cell.

## Figures and Tables

**Table 1:** Photovoltaic parameters of perovskite solar cells as a function of PbCl_2_ concentration [42]. Copyright © 2015, Elsevier, Amsterdam, The Netherlands.

Precursor Solutions (MA:Pb:I:Cl)	Performance Type	J_SC_ (mA/cm^2^)	V_OC_ (mV)	FF (%)	PCE (%)
1 M PbI_2_ + 1 M MAI (1:1:3:0)	Average	3.32 ± 1.87	649 ± 311	46.3 ± 12.6	1.40 ± 0.97
	Best	4.74	895	56.3	2.36
0.3 M PbCl_2_ + 0.7 M PbI_2_ + 1.6 M MAI (1.6:1:3:0.6)	Average	15.18 ± 2.55	843 ± 40	40.4 ± 5.4	5.19 ± 1.20
	Best	17.15	898	45.8	7.06
0.5 M PbCl_2_ + 0.5 M PbI_2_ + 2.0 M MAI (2:1:3:1)	Average	19.08 ± 1.65	979 ± 40	55.5 ± 4.1	10.41 ± 1.52
	Best	19.9	996	62.1	12.32
0.8 M PbCl_2_ + 0.2 M PbI_2_ + 2.6 M MAI (2.6:1:3:1.6)	Average	19.15 ± 0.88	965 ± 35	57.6 ± 4.0	10.68 ± 1.26
	Best	20.1	961	63.0	12.16
1 M PbCl_2_ + 3 M MAI (3:1:3:2)	Average	18.28 ± 1.16	857 ± 87	50.4 ± 5.4	7.95 ± 1.66
	Best	18.74	996	57.7	10.77

**Table 2:** Parameters of Devices Annealed under Different Atmospheric Conditions [115]. Copyright © 2015, American Chemical Society.

Solvent	Scan Direction	V_OC_ (V)	*J*_SC_ (mA/cm^2^)	FF (%)	PCE_max_ (%)	PCE_ave_ (%) ^1^
N_2_	Forward	0.93	16.9	0.53	8.34	7.39 ± 0.62
	Reverse	0.91	17.1	0.55	8.55	7.59 ± 0.60
H_2_O	Forward	0.95	18.7	0.51	8.99	7.50 ± 1.40
	Reverse	0.94	18.6	0.51	8.85	7.62 ± 1.14
γ-butyrolactone (GBL)	Forward	0.92	20.9	0.64	12.29	11.50 ± 0.74
	Reverse	0.92	20.8	0.65	12.47	11.32 ± 0.86
DMF	Forward	10.91	20.2	0.62	11.29	10.47 ± 0.82
	Reverse	0.91	20.3	0.64	11.89	10.65 ± 0.70
DMSO	Forward	0.93	20.9	0.68	13.21	11.89 ± 1.43
	Reverse	0.93	20.9	0.69	13.59	12.04 ± 1.27

^1^ Average PCE Values were obtained from 5 to 8 Cells for each type of devices.
